# Exploring the dynamics of adult *Axin2* cell lineage integration into dentate gyrus granule neurons

**DOI:** 10.3389/fnins.2024.1353142

**Published:** 2024-02-21

**Authors:** Khadijeh A. Sharifi, Faraz Farzad, Sauson Soldozy, Matthew R. DeWitt, Richard J. Price, Jason Sheehan, M. Yashar S. Kalani, Petr Tvrdik

**Affiliations:** ^1^Department of Neurological Surgery, University of Virginia Health System, Charlottesville, VA, United States; ^2^Department of Neuroscience, University of Virginia, Charlottesville, VA, United States; ^3^Department of Neurosurgery, Westchester Medical Center and New York Medical College, Valhalla, NY, United States; ^4^Department of Focused Ultrasound Cancer Immunotherapy Center, University of Virginia, Charlottesville, VA, United States; ^5^Biomedical Engineering, University of Virginia, Charlottesville, VA, United States; ^6^School of Medicine, St. John’s Neuroscience Institute, University of Oklahoma, Tulsa, OK, United States

**Keywords:** adult neurogenesis, dentate gyrus, Wnt signaling, *Axin2* cell lineage, focused ultrasound

## Abstract

The Wnt pathway plays critical roles in neurogenesis. The expression of *Axin2* is induced by Wnt/β-catenin signaling, making this gene a reliable indicator of canonical Wnt activity. We employed pulse-chase genetic lineage tracing with the *Axin2-CreERT2* allele to follow the fate of *Axin2*+ lineage in the adult hippocampal formation. We found *Axin2* expressed in astrocytes, neurons and endothelial cells, as well as in the choroid plexus epithelia. Simultaneously with the induction of *Axin2* fate mapping by tamoxifen, we marked the dividing cells with 5-ethynyl-2′-deoxyuridine (EdU). Tamoxifen induction led to a significant increase in labeled dentate gyrus granule cells three months later. However, none of these neurons showed any EdU signal. Conversely, six months after the pulse-chase labeling with tamoxifen/EdU, we identified granule neurons that were positive for both EdU and tdTomato lineage tracer in each animal. Our data indicates that *Axin2* is expressed at multiple stages of adult granule neuron differentiation. Furthermore, these findings suggest that the integration process of adult-born neurons from specific cell lineages may require more time than previously thought.

## Introduction

The adult hippocampus generates new neurons from radial glia-like neural stem cells (NSCs) that are slowly dividing, or quiescent. After activation they proliferate asymmetrically to generate intermediate progenitor cells (IPCs), which differentiate further into neuroblasts. These neuroblasts develop into immature neurons and subsequently into mature granule cells that form synaptic connections and become integrated into the hippocampal circuitry (Denoth-Lippuner and Jessberger, 2021).

It is widely accepted that the dentate gyrus undergoes continuous production and integration of granule cells well into adulthood (Kempermann et al., 2018). Postnatally generated granule cells undergo a controlled maturation and differentiation process, followed by integration into existing networks (Abrous et al., 2005). Although not completely understood, it is thought that immature granule cells work with mature granule cells to integrate sensory stimuli into context, allowing for subsequent contextual discrimination of recurrent similar stimuli (Tuncdemir et al., 2019). More recent work has allowed for recording of the *in vivo* activity of immature, adult born granule cells. These younger adult-born granule cells in the hippocampus appear to fire more often but show less spatial tuning specificity than mature granule cells (Danielson et al., 2016). Conversely, it has been proposed that adult-born granule cells transiently support sparser hippocampal population activity structure for effective mnemonic information processing (Mchugh et al., 2022). The adult-born neurons are also morphologically distinct from neonatally-born neurons (Cole et al., 2020). Notably, it is estimated that adult neurogenesis produces half of the granule cells in the mouse dentate gyrus (Cole et al., 2020).

Several signaling pathways, such as Hedgehog, Notch and Wnt, regulate various facets of neural stem cell (NSC) behavior (Alvarez-Buylla and Lim, 2004; Kriegstein and Alvarez-Buylla, 2009). Both in the CNS and in other tissues, Wnt signals act as morphogens in a concentration-dependent manner to control progenitor proliferation, tissue domains, and cell fate specification (Grigoryan et al., 2008; Wang et al., 2012). Moreover, canonical Wnt/β-catenin has a pivotal function in neuronal circuit formation (Inestrosa and Varela-Nallar, 2015). Specifically, *Axin2* is one of the first genes induced by canonical Wnt signals, and it has proved to be a valuable genetic tool in the Wnt research field. Wnt/β-catenin-responsive cells have been tracked *in vivo* in many tissues and disease contexts using an *Axin2-CreERT2* allele model (Van Amerongen et al., 2012). This tamoxifen inducible line has been also used to detect neurogenesis and trace neuronal lineages *in vivo* (Bowman et al., 2013).

The time required for neural stem cells to mature and integrate fully into existing neural networks is still a subject of debate. In 2011, Encinas and coworkers reported that quiescent neural progenitors in the mouse dentate gyrus develop into fully mature cells through multiple controlled and regulated divisions (Encinas et al., 2011). They showed that this entire maturation process, from quiescent neuronal precursors to mature new cells, takes approximately 1 month. More recently, Goncalves et al. reported that these new cells form functional connections within 2–3 weeks after their last mitosis, thus integrating into already established networks more quickly than previously reported (Goncalves et al., 2016).

In order to further advance our current understanding of the role that the Wnt/β-catenin pathway plays in adult neurogenesis in the dentate gyrus, we conducted a neuronal lineage tracing experiment to measure the proliferation of *Axin2+* lineage in a pulse-chase manner. We show that the first evidence of mature granule cells derived from adult neurogenesis is seen 6 months after the initial labeling with *Axin2-CreERT2*. We propose that different cell lineages might have a different pace of integration during adult neurogenesis.

## Materials and methods

### Animals and treatments

The *Axin2^CreERT2^* mice (Bowman et al., 2013) (Strain #: 018867, RRID: IMSR_JAX:018867) were purchased from JAX Mice (The Jackson Laboratory, Bar Harbor, ME) and bred in-house. The PC::G5-tdT line (Gee et al., 2014) is maintained in our laboratory. All experiments were carried out in 10-12-week-old mice at the time of induction. All animals were fed standard rodent chow and housed under 12-h light/12-h dark cycle. Four groups of 3 mice were analyzed, for a total of 24 bilateral hippocampal samples. All mice received tamoxifen (100 mg/kg; i.p.) and 5-ethynyl-2′-deoxyuridine (EdU) (40 mg/kg body weight; i.p.) on day *t* = 0. The animals were sacrificed and assessed after 7, 30, 90 and 180 days, based on random assignment to the pulse-chase group.

### Histology and immunohistochemistry

Mice were transcardially perfused with PBS and 4% phosphate-buffered formaldehyde. Brains were then dissected, post-fixed in 4% PFA and equilibrated in 10 and 30% sucrose, followed by embedding in OCT. Frozen brains were sectioned coronally at 25-μm thickness and stored at −20°C. For staining, the sections were rehydrated with PBS and permeabilized with 0.1% Triton X-100 (Sigma, United States) for 1 h at room temperature. Next, the slides were incubated for 30 min at room temperature and processed with Click-iT™ EdU Alexa Fluor™ 488 Imaging kit (Fisher Scientific), followed by after washes with Triton X-100 in PBS. Next, immunostaining was performed with rabbit anti-Red Fluorescent Protein (RFP, 1:500, Rockland, United States), guinea pig anti-doublecortin (DCX, 1:500, Millipore Sigma, USA); mouse anti-glial fibrillary acidic protein (GFAP, 1:1000, Millipore Sigma, United States), anti-NeuN (1:500, Millipore Sigma, United States), anti-Iba1 (Wako) and anti-Transthyretin (abcam). Secondary antibodies included donkey anti-rabbit Alexa 568 (1:500, Molecular Probes, Invitrogen); goat anti-guinea pig Alexa 647 (1:500, Abcam, ab150187); donkey anti-mouse Alexa 647 (1:500, Molecular Probes, Invitrogen). Slides were mounted with ProLong™ Gold antifade (Invitrogen, United States).

### Confocal microscopy and image analysis

Fluorescence signals were imaged with Zeiss LSM-880 with Airyscan confocal microscope (Zeiss, Germany) using sequential scanning mode for Alexa 405, 488, 568 and 647. Stacks of images (1,024 × 1,024 pixels) were tiled across the dentate gyrus area. Dentate gyri were segmented in the tiled 3D datasets with Imaris 9 (Bitplane, Oxford), and the cells labeled with specific antibodies were detected and counted within the segmented dentate gyrus using the Spots model. Total cell counts in the segmented regions were then normalized per 1,000 DAPI-positive nuclei in the dentate gyrus.

### Statistical analysis

The results were expressed as Mean ± standard deviation (SD). In all experiments, the statistical significance was set at *p* < 0.05. Calculations were performed with one-way ANOVA with post-hoc Tukey HSD test, or post-test for liner trend, using the statistical package software Prism 6 (GraphPad, San Diego, CA).

## Results

### *Axin2^CreERT2^* genetically labels neuronal, glial and endothelial cells in the dentate gyrus

**Figure 1:**
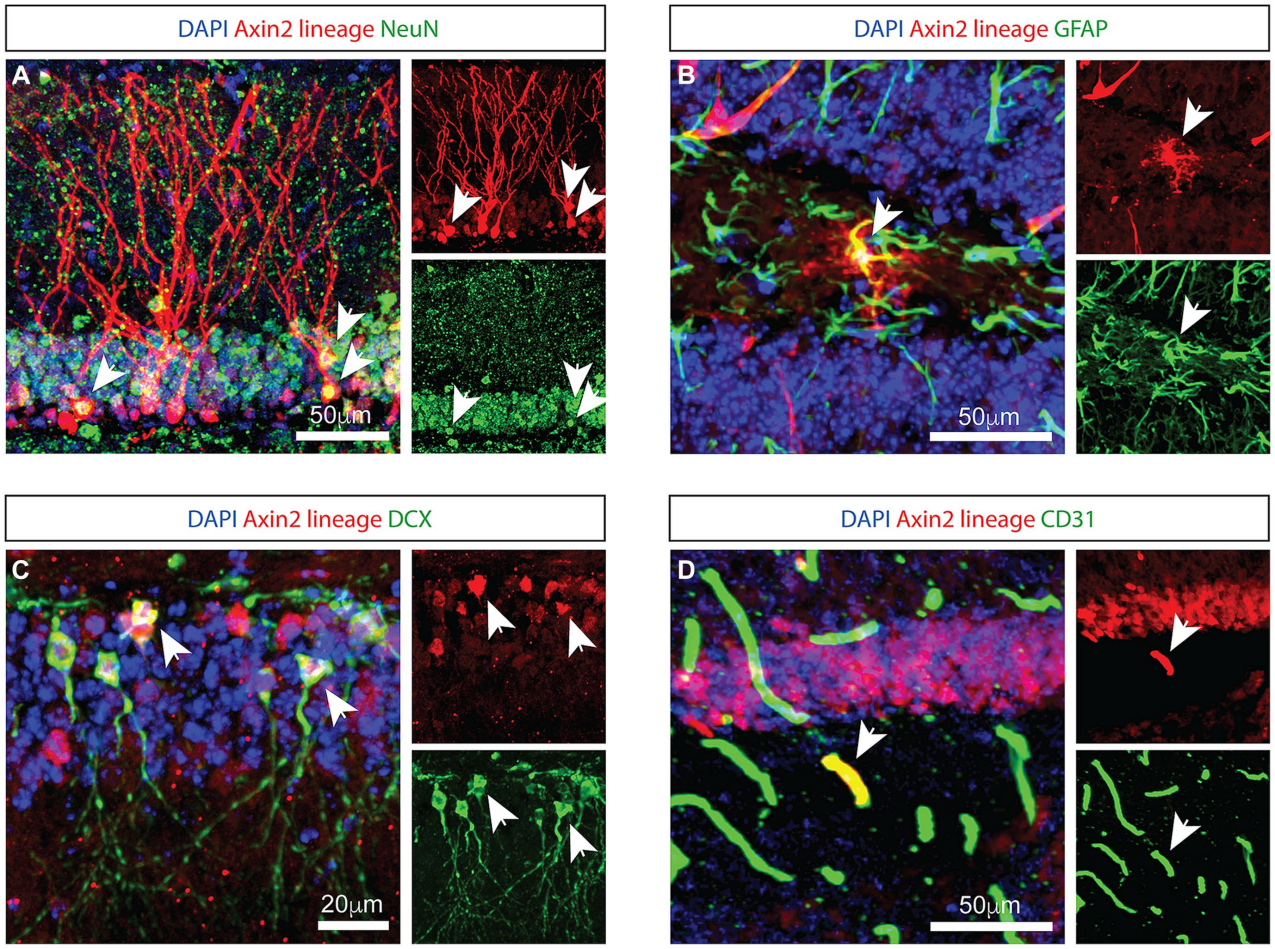
Genetic labeling with *Axin2* ^CreERT2^ reveals neural, astrocytic, and endothelial cells. **(A)** *Axin2*-labeled tdTomato-positive neurons in the granule layer of the dentate gyrus are NeuN-positive. Arrowheads identify corresponding neuronal cell bodies in the 3-channel overlay (left), as well as in the specific channels showing tdTomato (upper right) and NeuN (lower right). **(B)** *Axin2*-labeled astrocytes in the dentate gyrus region co-stain with GFAP antibodies. **(C)** Co-staining with doublecortin (DCX) identifies neuroblasts positive for *Axin2* lineage markers. **(D)** *Axin2^CreERT2^* also labels a subset of endothelial cells in the dentate gyrus region which react with antibodies to CD31. In all panels, white arrows identify double-positive cells. Scale bars, 50 μm, unless indicated otherwise.

To identify *Axin2*-expressing cells in the hippocampus, we crossed *Axin2^CreERT2^* mice (Van Amerongen et al., 2012) to the reporter line expressing tdTomato following Cre recombination (PC::G5-tdT) (Gee et al., 2014), and analyzed the progeny with immunohistochemistry. Previous reports induced the *Axin2^CreERT2^* allele in embryonic and juvenile mice (Bowman et al., 2013). Consistent, adult-induced lineage labeled similar cell types in the hippocampus, one week after a single injection of tamoxifen (Figure 1). In the granule layer of dentate gyrus, a subset of *Axin2*-lineage-positive granule neurons was observed with mature appearing dendrites in the molecular layer. These cells co-labeled with the neuronal marker NeuN (Figure 1A). In the sub granular zone, GFAP-positive, ramified cells were observed with a characteristic appearance of astrocytes (Figure 1B). *Axin2*+ lineage cells lacking dendritic morphology were frequently detected in the subgranular zone. Some of these cells co-stained with doublecortin (DCX), a marker of immature neurons (La Rosa et al., 2020) (Figure 1C). We also show that *Axin2* is expressed in endothelial cells on a subset of blood vessels co-staining with PECAM1/CD31 (Figure 1D). Of note, in the third ventricle choroid plexus adjacent to hippocampus, we have also detected *Axin2*+ lineage in the choroid epithelia, but not in myeloid cells (Supplementary Figure S1). Together, *Axin2* is expressed in several cell types in the hippocampal neurovascular unit, showing increased density in the subgranular zone.

### Dynamics of the labeled *Axin2+* cell population in the granular layer

**Figure 2:**
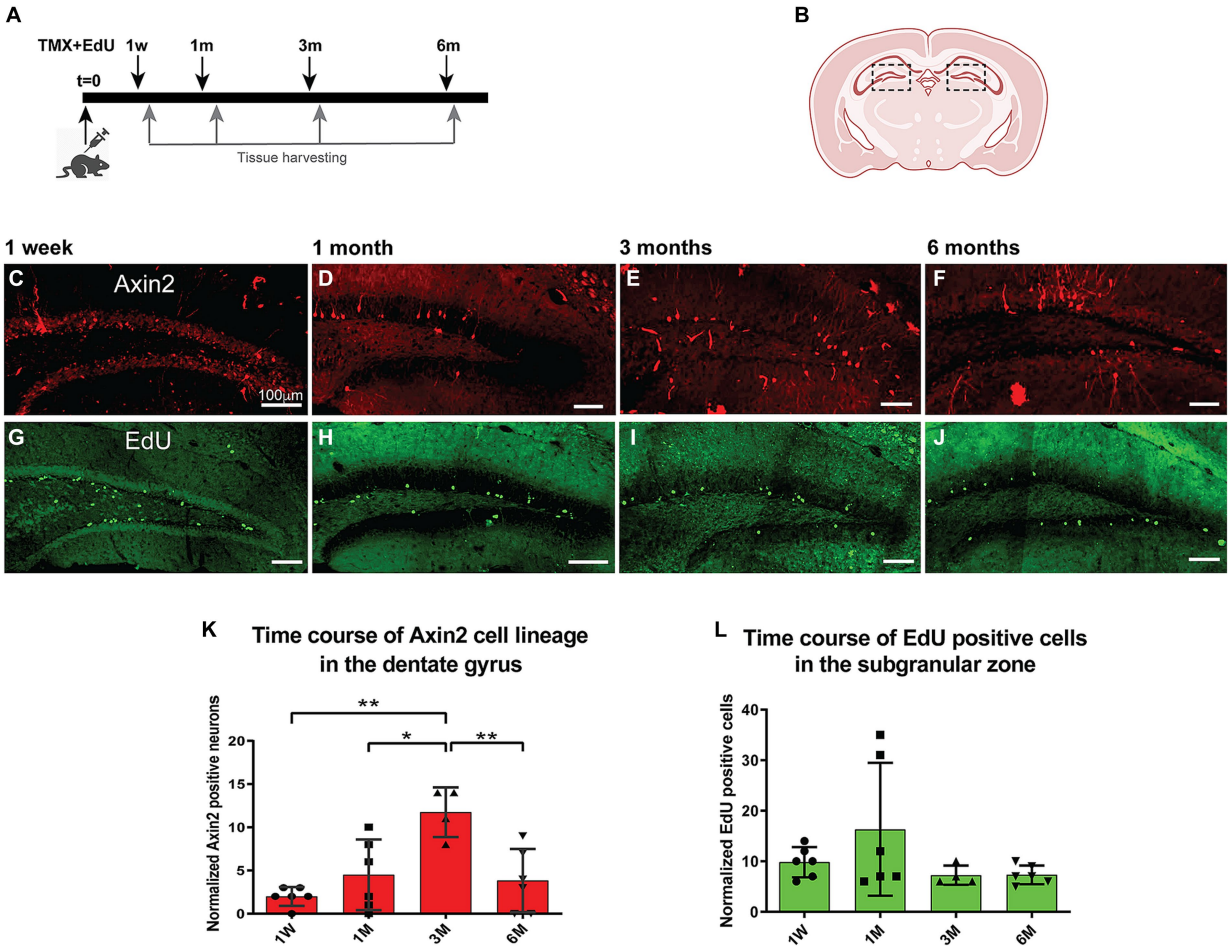
Time course of adult induced *Axin2* cell population in the dentate gyrus. **(A)** Schematic diagram showing the pulse-chase strategy used in this study. Adult 12-wk-old animals were induced with tamoxifen and concurrently injected with a single dose of EdU. The brains were analyzed at 1 week (1 W), 1 month (1 M), 3 months (3 M) and 6 months (6 M) post induction. **(B)** The central coronal plane used as a landmark for sectioning. The dashed boxes highlight the dentate gyrus (DG) area analyzed in this study. **(C–F)** Representative confocal images showing tdTomato signal reporting *Axin2* cell lineage. **(G–J)** Typical EdU staining in DG at different chase time points. The bulk of staining moved from subgranular layer to the deeper granular layer of the DG. **(K)** Graph showing temporal dynamics of the *Axin2* cell lineage. **(L)** Time course of EdU-positive cell density during the chase period (1w – 6 m). Counts in both plots were normalized per 1,000 DAPI positive nuclei. *N* = 2–3 mice and 12–18 sections for each time point. For each brain, three sections were analyzed. The ipsilateral and contralateral sides from all section were combined and averaged, resulting in two mean values for each animal. ^*^p < 0.05, **p < 0.01. Scale bars, 100 μm.

**Figure 3:**
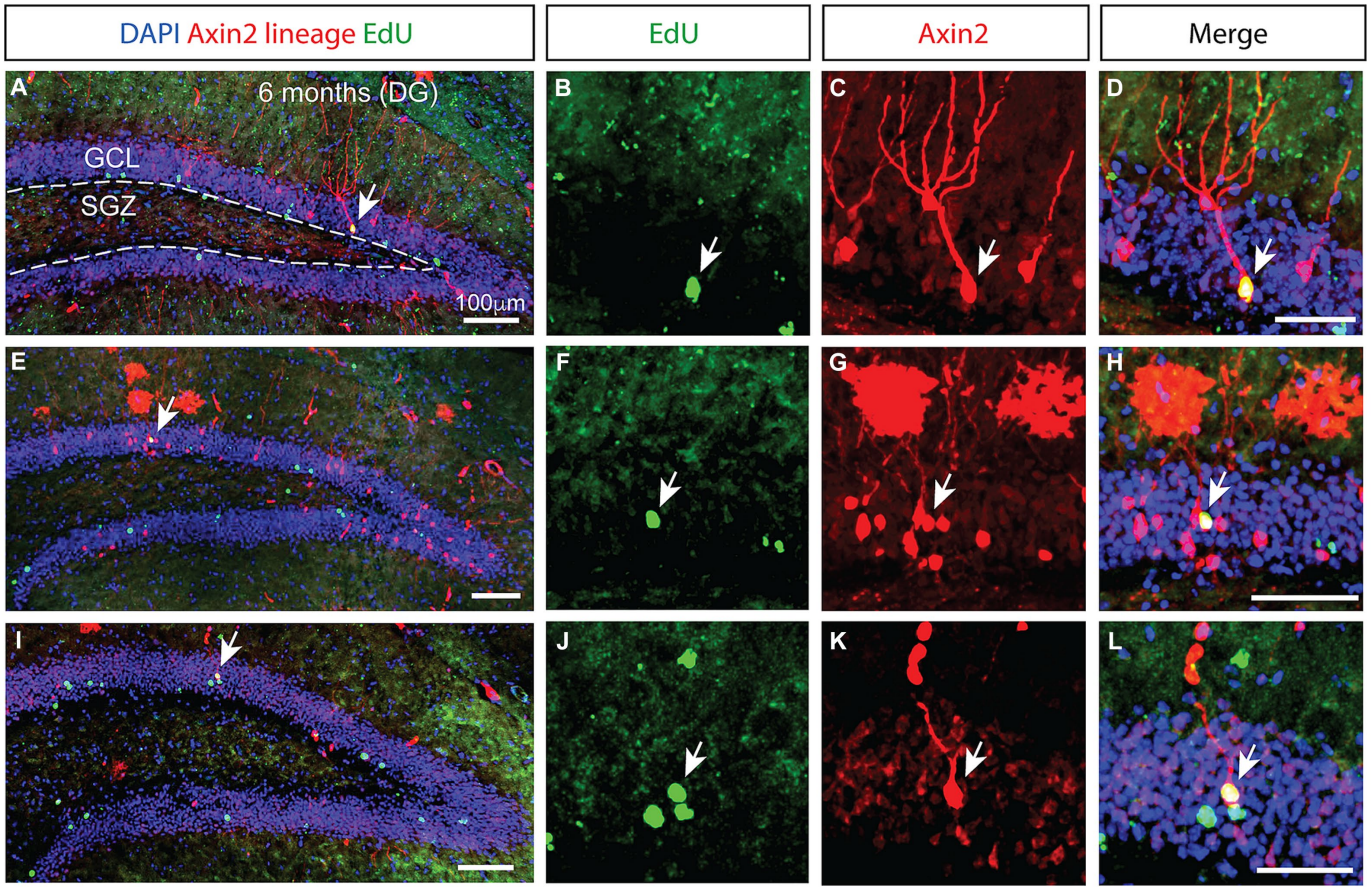
Prolonged integration of mitotically labeled *Axin2*-positive cell lineage in the granular layer of the dentate gyrus. **(A,E,I)** Low magnification confocal images of dentate gyrus sections from three different animals 6 months after tamoxifen induction. The staining shows *Axin2* cell lineage (red), EdU (green) and DAPI (blue). **(A)** The granular cell layer (GCL) is outlined with white dashed lines, and the location of the subgranular zone (SGZ) is indicated. Arrows pinpoint the *Axin2*- and EdU-positive cells. **(B,F,J)** Higher magnification panels showing the EdU signal in the granule cell layer. **(C,G,K)** Matching panels showing the tdTomato signal indicating *Axin2* cell lineage in the same region as **(B,F,J)**. **(D,H,L)** Overlay of EdU and *Axin2* panels with DAPI signal, highlighting the mitotically labeled granule neurons originating from the *Axin2* lineage. Low magnification scale bars, 100 μm. High magnification scale bars, 50 μM. DG, dentate gyrus; GCL, granular cell layer; SGZ, subgranular zone.

To follow the fate of *Axin2+* lineage in dentate gyrus neurogenesis over time, we performed a time course analysis of granule cells labeled with the tracer tdTomato. Adult mice were induced with tamoxifen (100 mg/kg) and followed over time from 1 week to 6 months (Figure 2A). Differentiated neurons with mature dendrites in the dentate gyrus were counted and normalized per 1,000 DAPI-positive nuclei detected with image analysis (Figures 3A,E,I; Methods). Our analysis revealed a statistically significant 6-fold increase (from 2.0 ± 1.1 after 1 week, to 12.0 ± 3.7 after 3 months) in the density of *Axin2* granular cells in the animals sacrificed 90 days after tamoxifen injection when compared to the 1-week time point (Figure 2K). There was also a statistically significant positive linear trend for the first 3 months (*p* = 0.0432, ANOVA, post-test for trend). The *Axin2*-lineage-positive granule cell population peaked around 3 months, but subsequently decreased by 6 months (Figure 2). It is also noteworthy that the *Axin2^CreERT2^* line demonstrates baseline leakiness of recombination. As a result, a small number of cells, including granule neurons, become labeled with the tracer even without tamoxifen induction (Supplementary Figure S2). To compensate for this background leakiness, we have measured the number of false-positive granule neurons in three animals and subtracted the normalized and averaged counts from the nominal values determined at all chase time points in this study, as described in the Methods and Supplementary Figure S2.

### The time course of aggregate EdU labeling in the dentate gyrus

Next, we investigated cell proliferation in the *Axin2* cell lineage. We performed EdU pulse-chase experiments concurrent with the induction of *Axin2^CreERT2^* labeling. The animals were injected with a single dose of EdU (40 mg/kg, i.p.) concurrently with tamoxifen, and analyzed at the same four time points from 1 week to 6 months (Figures 2G–J). Edu-positive cells were detected and measured in the subgranular zone and granular zone, and the values were normalized per DAPI-stained 1,000 nuclei (Figure 2L). After 1 month, the number of EdU-labeled cells increased 1.5-fold, but this change was not significant. The numbers of EdU-positive cells remained stable at later time points at approximately 7.3 ± 1.9 EdU-positive nuclei per 1,000 DAPI-positive nuclei.

### Delayed emergence of Edu-positive *Axin2+* granule neurons in the dentate gyrus

**Figure 4:**
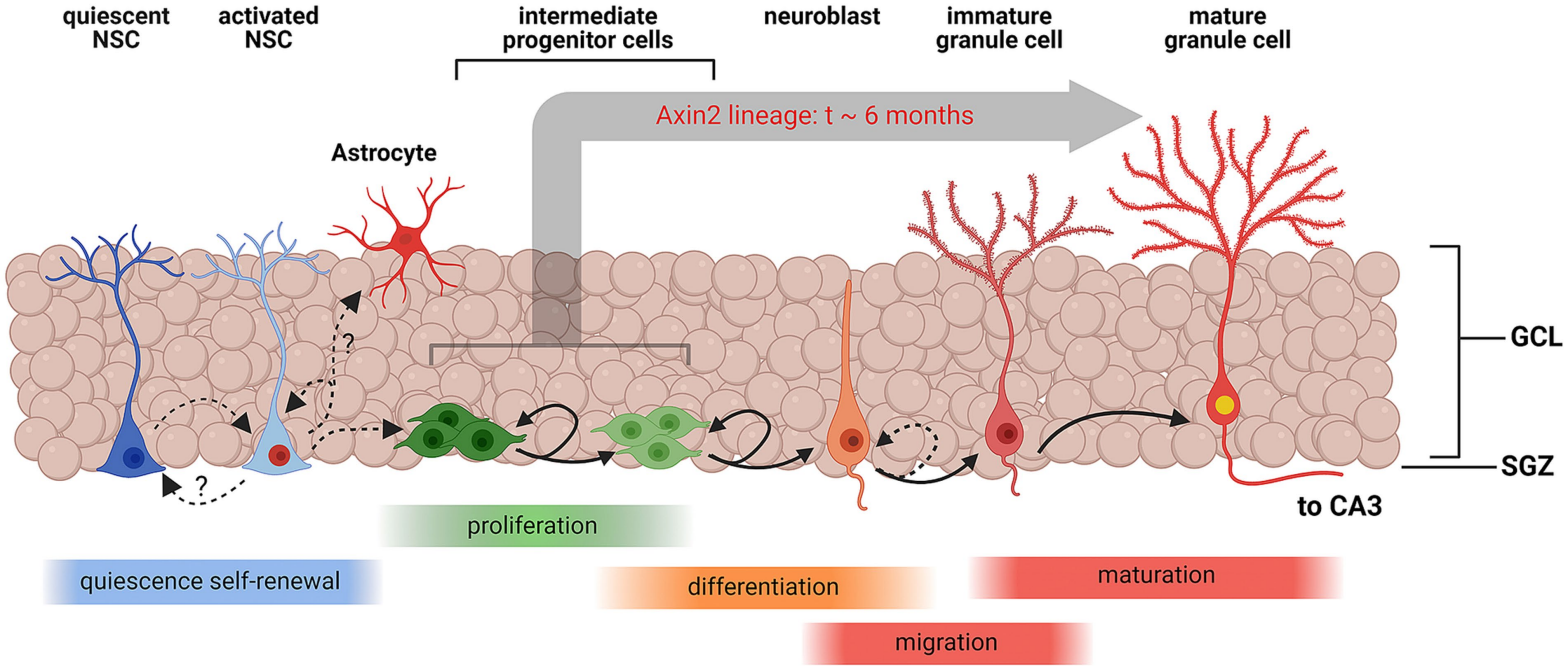
Schematic representation of Wnt-associated subgranular neurogenesis. A summary diagram of the stages in subgranular neurogenesis from quiescent neuronal stem cells (NSCs) to mature granule cells. Quiescent NSCs are multipotent undifferentiated cells. Once induced, quiescent NSCs differentiate into activated NSCs, which may replenish the pool of quiescent NSCs, or commit to the astrocyte maturation pathway. Activated NSCs also develop into multipolar intermediate progenitor cells and neuroblasts which continue to proliferate and eventually migrate inside the granular layer and enter the granule cell maturation pathway. *Axin2*-positive NSCs and progenitors represent a sub-lineage of the cell lines, contributing to subgranular neurogenesis. In this study, we have unequivocally detected *Axin2* lineage markers in the intermediate progenitors and neuroblasts, which are amenable to mitotic labeling, and post-mitotic mature neurons. Our data suggest that the process of integration of progenitors into mature granule cells can take as long as 6 months in this cell lineage. Created with BioRender.com.

While the number of *Axin2*-lineage-positive granule neurons peaked 3 months following the induction, we found no ramified, differentiated neurons containing EdU marker at this stage. However, in the animals analyzed 6 months after the initial pulse, double positive neurons containing the *Axin2* lineage markers as well as the EdU label were detected (Figure 3), indicating their derivation from the Wnt-dependent progenitor pool. The neuronal bodies of these double-positive cells were located in the central part of the granular layer (Figures 3H,L). Additional *Axin2*-negative nuclei labeled with EdU were seen in the granular layer (Figure 3L), consistent with neuronal identity. Together, our findings confirm that Wnt-dependent *Axin2* cell lineage in the adult brain gives rise to a subset of dentate gyrus granule neurons. Surprisingly, the timeline of maturation and integration of these neurons is considerably longer than what is generally accepted as the time interval needed for neuron differentiation during the adult neurogenesis (Figure 4).

## Discussion

We have characterized the cell fate of the adult induced *Axin2* cell lineage. While others have previously shown that *Axin2* expression is active in hippocampus (Bowman et al., 2013), the genetic labeling in their study was induced at the embryonic and juvenile stages within first two postnatal weeks of development. Our experiments confirm that *Axin2* plays role in the neurogenesis of granule cells of the adult dentate gyrus. Consistent, *Axin2* labeling was observed in DCX-positive immature neurons. Conversely, we have not identified *Axin2* in NSC or IPC cells, and more precise characterization of the *Axin2* progenitor population is warranted. *Axin2* expression has been previously reported in astrocytes (Kalani et al., 2008). It is also possible that these tdTomato-labeled astrocytes are born from the *Axin2*-positive NSCs (Figure 4), and thus derived from the *Axin2* domain in NSC. We have noticed larger counts of tdTomato-labeled astrocytes at 3- and 6-month pulse-chase time points, but these increases were not formally analyzed. Furthermore, we have also shown that *Axin2* expression is present in microvascular endothelia, in line with the role of Wnt/β-catenin signaling in angiogenesis and vascular integrity (Daneman et al., 2009; Dejana, 2010; Foulquier et al., 2018; Liebner et al., 2018).

In addition, *Axin2* is expressed in the choroid plexus. We found that the *Axin2* cell lineage did not label any myeloid cells, neither microglia nor macrophages. Instead, these cells co-stained with Transthyretin, a marker of choroid plexus epithelial cells (Supplementary Figures S1E–H; Vancamp et al., 2019). The choroid plexus generates cerebrospinal fluid, and the epithelia distributes thyroid hormone and secretes numerous factors, including Wnt proteins. Thus, the *Axin2*-lineage of choroid plexus can potentially affect cerebral proliferative processes in a long-range manner (Kaiser et al., 2019; Vancamp et al., 2019).

The *Axin2+* granule neurons presented a dynamic cell population in our experiments. Our measurements imply that the *Axin2-*lineage*-*positive granule cell population is replenished from the *Axin2*-labeled progenitors and increases six-fold after 3 months. Interestingly, our work also showed that the *Axin2-*lineage*-*positive granule cells subsequently decreased between 3 months and 6 months. This suggests that these neurons may have a limited lifespan, but additional research is required to explore this possibility.

Encinas et al. have demonstrated that both NSCs and amplifying IPCs decrease with age in mouse lines (Encinas et al., 2011). Yet here we show that the *Axin2* arm of the canonical Wnt pathway remains active in the adult dentate gyrus neurogenic niche. Along with other neurogenic factors, like sonic hedgehog (Shh), bone morphogenetic proteins (BMPs), and Notch, Wnt ligands are known regulators of adult hippocampal neurogenesis (Inestrosa and Varela-Nallar, 2015; Urbach and Witte, 2019). Wnts are expressed by local astrocytes and NSCs themselves, acting as paracrine and autocrine factors in the NSC niche (Lie et al., 2005; Wexler et al., 2009). Specific ligands such as Wnt3 have been shown to promote neuroblast proliferation and neuronal differentiation through the canonical Wnt pathway (Lie et al., 2005). In addition, Wnt signaling also emerged as important pathway promoting multipotency and self-renewal of NSCs (Mao et al., 2009). However, the specific roles of *Axin2* in these processes remain to be characterized.

One important question to address is whether different subsets of NSCs exist with various degrees of quiescence and distinct abilities to generate neurons and glial cells. Genetic lineage tracing with inducible version of the Cre recombinase under different promoters has provided evidence for the functional heterogeneity of NSCs in the dentate gyrus (Decarolis et al., 2013; Urban et al., 2019). Some NSCs have been shown to be neurogenic and short lived, while others are multipotent and self-renew for longer periods (Ibrayeva et al., 2021). Using genetic labeling with nestin-CreER and performing a double pulse-chase experiment with tamoxifen and BrdU, Encinas detected BrdU- and NeuN-positive granule neurons in the nestin cell lineage 30 days after pulse-chase induction (Encinas et al., 2011). This is in stark contrast with our results suggesting that *Axin2* cell lineage requires much longer for neuronal differentiation and integration into granule neurons. Since *Axin2^CreERT2^* labels fewer neuronal progenitors than nestin-CreER, some co-labeled cells could have been missed. It is surprising, however, that in comparison to the 6-month time point, twice as many *Axin2*+ lineage cells were seen after the 3-month pulse-chase, with a similar general level of EdU staining, yet no tdTomato+ EdU+ neurons were detected (Figures 3K,L). The underrepresentation of EdU-positive neurons at earlier time points may be partially due to dilution of the EdU label in progenitor cells. Conversely, it is unlikely but not entirely impossible that the observed tdTomato labeling in the adult EdU-positive granule cells results from nonspecific activation by the leaky *Axin2^CreERT2^* driver.

We also acknowledge that the genetic labeling approach used in this study was rather sparse, consisting of a single simultaneous injection of tamoxifen and EdU. Designed to facilitate effective lineage tracing, the resulting labeling density did not provide sufficient labeling of stem cells and neuronal progenitors, as stated above. More saturating labeling strategies will have to be employed in future studies to reveal the presence of *Axin2* in quiescent adult neural stem cells. During the completion of this study, Luo et al. (2023) conducted a comprehensive study comparing *Axin2^CreERT2^* and *Gli1^CreERT2^* expression in neural stem cells of the adult brain (Luo et al., 2023). The authors observed *Axin2^CreERT2^* activity in both quiescent and activated stem cells 1 month after a single tamoxifen injection at 3, 6, and 12 months. Furthermore, they found a higher number of activated *Axin2+* clones compared to *Gli1+* clones 1 month after tamoxifen injection, suggesting that *Axin2+* adult dentate NSCs are more likely to sustain neurogenesis over time, while *Gli1+* adult dentate NSCs tend to become quiescent with age and contribute to the decline in age-induced neurogenesis. However, it should be noted that the leakiness of the *Axin2^CreERT2^* genetic system, demonstrated in our study, may have influenced their measurements.

A potential direction for future research is to explore whether neurogenesis in the aging brain can be safely and non-invasively stimulated. One emerging opportunity is to use focused ultrasound. Focused ultrasound (FUS) is a non-invasive technique that can be used to precisely target regions in the brain for various therapeutic purposes including thermal ablation and drug delivery (Meng et al., 2021). When combined with systemically delivered, gas-filled microbubbles, FUS can safely and transiently increase the permeability of the blood–brain barrier (BBB) to facilitate the delivery of therapeutic agents. Recent reports have demonstrated that FUS mediated BBB opening can induce hippocampal neurogenesis in adult mice, independent of delivery of therapeutic agents (Scarcelli et al., 2014). While the mechanism remains to be elucidated, the neurogenic effect is likely due to localized increases in BDNF, VEGF, and bFGF expression following FUS delivery (Shin et al., 2019) and only in the presence of transient BBB opening (Mooney et al., 2016). Future experiments investigating the relationship between opening BBB, stimulating the Wnt/β-catenin pathway, and utilizing the *Axin2^CreERT2^* cell lineage as a readout could provide valuable insights into the potential for FUS-mediated neurogenesis in various neurodegenerative disorders.

## Data availability statement

The original contributions presented in the study are included in the article/Supplementary material, further inquiries can be directed to the corresponding authors.

## Ethics statement

The animal study was approved by the Institutional Animal Care and Use Committee (IACUC) at the University of Virginia. The study was conducted in accordance with the local legislation and institutional requirements.

## Author contributions

KS: Conceptualization, Data curation, Formal analysis, Investigation, Methodology, Software, Supervision, Visualization, Writing – original draft. FF: Data curation, Formal analysis, Investigation, Methodology, Software, Visualization, Writing – original draft. SS: Conceptualization, Investigation, Methodology, Writing – original draft. MD: Investigation, Methodology, Writing - review & editing. RP: Conceptualization, Funding acquisition, Resources, Writing – review & editing. JS: Resources, Investigation, Writing - review & editing. MK: Conceptualization, Funding acquisition, Project administration, Resources, Supervision, Writing – review & editing. PT: Conceptualization, Writing – original draft, Writing – review & editing, Funding acquisition, Methodology, Project administration, Resources, Software, Supervision.

## References

[ref1] Abrous D. N. Koehl M. Le Moal M. (2005). Adult neurogenesis: from precursors to network and physiology. Physiol. Rev. 85, 523–569. doi: 10.1152/physrev.00055.2003 15788705

[ref2] Alvarez-Buylla A. Lim D. A. (2004). For the long run: maintaining germinal niches in the adult brain. Neuron 41, 683–686. doi: 10.1016/S0896-6273(04)00111-4, PMID: 15003168

[ref3] Bowman A. N. Van Amerongen R. Palmer T. D. Nusse R. (2013). Lineage tracing with Axin2 reveals distinct developmental and adult populations of Wnt/beta-catenin-responsive neural stem cells. Proc. Natl. Acad. Sci. USA 110, 7324–7329. doi: 10.1073/pnas.1305411110, PMID: 23589866 PMC3645553

[ref4] Cole J. D. Espinueva D. F. Seib D. R. Ash A. M. Cooke M. B. Cahill S. P.. (2020). Adult-born hippocampal neurons undergo extended development and are morphologically distinct from Neonatally-born neurons. J. Neurosci. 40, 5740–5756. doi: 10.1523/JNEUROSCI.1665-19.2020, PMID: 32571837 PMC7380968

[ref5] Daneman R. Agalliu D. Zhou L. Kuhnert F. Kuo C. J. Barres B. A. (2009). Wnt/beta-catenin signaling is required for CNS, but not non-CNS, angiogenesis. Proc. Natl. Acad. Sci. USA 106, 641–646. doi: 10.1073/pnas.0805165106 19129494 PMC2626756

[ref6] Danielson N. B. Kaifosh P. Zaremba J. D. Lovett-Barron M. Tsai J. Denny C. A.. (2016). Distinct contribution of adult-born hippocampal granule cells to context encoding. Neuron 90, 101–112. doi: 10.1016/j.neuron.2016.02.019, PMID: 26971949 PMC4962695

[ref7] Decarolis N. A. Mechanic M. Petrik D. Carlton A. Ables J. L. Malhotra S.. (2013). In vivo contribution of nestin- and GLAST-lineage cells to adult hippocampal neurogenesis. Hippocampus 23, 708–719. doi: 10.1002/hipo.22130, PMID: 23554226 PMC3732558

[ref8] Dejana E. (2010). The role of wnt signaling in physiological and pathological angiogenesis. Circ. Res. 107, 943–952. doi: 10.1161/CIRCRESAHA.110.223750 20947863

[ref9] Denoth-Lippuner A. Jessberger S. (2021). Formation and integration of new neurons in the adult hippocampus. Nat. Rev. Neurosci. 22, 223–236. doi: 10.1038/s41583-021-00433-z 33633402

[ref10] Encinas J. M. Michurina T. V. Peunova N. Park J. H. Tordo J. Peterson D. A.. (2011). Division-coupled astrocytic differentiation and age-related depletion of neural stem cells in the adult hippocampus. Cell Stem Cell 8, 566–579. doi: 10.1016/j.stem.2011.03.010, PMID: 21549330 PMC3286186

[ref11] Foulquier S. Daskalopoulos E. P. Lluri G. Hermans K. C. M. Deb A. Blankesteijn W. M. (2018). WNT signaling in cardiac and vascular disease. Pharmacol. Rev. 70, 68–141. doi: 10.1124/pr.117.013896, PMID: 29247129 PMC6040091

[ref12] Gee J. M. Smith N. A. Fernandez F. R. Economo M. N. Brunert D. Rothermel M.. (2014). Imaging activity in neurons and glia with a Polr2a-based and cre-dependent GCaMP5G-IRES-tdTomato reporter mouse. Neuron 83, 1058–1072. doi: 10.1016/j.neuron.2014.07.024, PMID: 25155958 PMC4156920

[ref13] Goncalves J. T. Schafer S. T. Gage F. H. (2016). Adult neurogenesis in the Hippocampus: from stem cells to behavior. Cell 167, 897–914. doi: 10.1016/j.cell.2016.10.021 27814520

[ref14] Grigoryan T. Wend P. Klaus A. Birchmeier W. (2008). Deciphering the function of canonical Wnt signals in development and disease: conditional loss- and gain-of-function mutations of beta-catenin in mice. Genes Dev. 22, 2308–2341. doi: 10.1101/gad.1686208, PMID: 18765787 PMC2749675

[ref15] Ibrayeva A. Bay M. Pu E. Jorg D. J. Peng L. Jun H.. (2021). Early stem cell aging in the mature brain. Cell Stem Cell 28:955:966. doi: 10.1016/j.stem.2021.03.018 33848469 PMC10069280

[ref16] Inestrosa N. C. Varela-Nallar L. (2015). Wnt signalling in neuronal differentiation and development. Cell Tissue Res. 359, 215–223. doi: 10.1007/s00441-014-1996-4 25234280

[ref17] Kaiser K. Gyllborg D. Prochazka J. Salasova A. Kompanikova P. Molina F. L.. (2019). WNT5A is transported via lipoprotein particles in the cerebrospinal fluid to regulate hindbrain morphogenesis. Nat. Commun. 10:1498. doi: 10.1038/s41467-019-09298-4, PMID: 30940800 PMC6445127

[ref18] Kalani M. Y. Cheshier S. H. Cord B. J. Bababeygy S. R. Vogel H. Weissman I. L.. (2008). Wnt-mediated self-renewal of neural stem/progenitor cells. Proc. Natl. Acad. Sci. USA 105, 16970–16975. doi: 10.1073/pnas.0808616105, PMID: 18957545 PMC2575225

[ref19] Kempermann G. Gage F. H. Aigner L. Song H. Curtis M. A. Thuret S.. (2018). Human adult neurogenesis: evidence and remaining questions. Cell Stem Cell 23, 25–30. doi: 10.1016/j.stem.2018.04.004, PMID: 29681514 PMC6035081

[ref20] Kriegstein A. Alvarez-Buylla A. (2009). The glial nature of embryonic and adult neural stem cells. Annu. Rev. Neurosci. 32, 149–184. doi: 10.1146/annurev.neuro.051508.135600, PMID: 19555289 PMC3086722

[ref21] La Rosa C. Parolisi R. Bonfanti L. (2020). Brain structural plasticity: from adult neurogenesis to immature neurons. Front. Neurosci. 14:75. doi: 10.3389/fnins.2020.00075, PMID: 32116519 PMC7010851

[ref22] Lie D. C. Colamarino S. A. Song H. J. Desire L. Mira H. Consiglio A.. (2005). Wnt signalling regulates adult hippocampal neurogenesis. Nature 437, 1370–1375. doi: 10.1038/nature04108 16251967

[ref23] Liebner S. Dijkhuizen R. M. Reiss Y. Plate K. H. Agalliu D. Constantin G. (2018). Functional morphology of the blood-brain barrier in health and disease. Acta Neuropathol. 135, 311–336. doi: 10.1007/s00401-018-1815-1, PMID: 29411111 PMC6781630

[ref24] Luo X. Dai M. Wang M. Wang X. Guo W. (2023). Functional heterogeneity of Wnt-responsive and hedgehog-responsive neural stem cells in the murine adult hippocampus. Dev. Cell 58, 2545–2562. doi: 10.1016/j.devcel.2023.07.021 37607545

[ref25] Mao Y. Ge X. Frank C. L. Madison J. M. Koehler A. N. Doud M. K.. (2009). Disrupted in schizophrenia 1 regulates neuronal progenitor proliferation via modulation of GSK3beta/beta-catenin signaling. Cell 136, 1017–1031. doi: 10.1016/j.cell.2008.12.044, PMID: 19303846 PMC2704382

[ref26] Mchugh S. B. Lopes-Dos-Santos V. Gava G. P. Hartwich K. Tam S. K. E. Bannerman D. M.. (2022). Adult-born dentate granule cells promote hippocampal population sparsity. Nat. Neurosci. 25, 1481–1491. doi: 10.1038/s41593-022-01176-5, PMID: 36216999 PMC9630129

[ref27] Meng Y. Hynynen K. Lipsman N. (2021). Applications of focused ultrasound in the brain: from thermoablation to drug delivery. Nat. Rev. Neurol. 17, 7–22. doi: 10.1038/s41582-020-00418-z, PMID: 33106619

[ref28] Mooney S. J. Shah K. Yeung S. Burgess A. Aubert I. Hynynen K. (2016). Focused ultrasound-induced neurogenesis requires an increase in blood-brain barrier permeability. PLoS One 11:e0159892. doi: 10.1371/journal.pone.0159892, PMID: 27459643 PMC4961388

[ref29] Scarcelli T. Jordao J. F. O'reilly M. A. Ellens N. Hynynen K. Aubert I. (2014). Stimulation of hippocampal neurogenesis by transcranial focused ultrasound and microbubbles in adult mice. Brain Stimul. 7, 304–307. doi: 10.1016/j.brs.2013.12.012, PMID: 24629831 PMC4103630

[ref30] Shin J. Kong C. Lee J. Choi B. Y. Sim J. Koh C. S.. (2019). Focused ultrasound-induced blood-brain barrier opening improves adult hippocampal neurogenesis and cognitive function in a cholinergic degeneration dementia rat model. Alzheimers Res. Ther. 11:110. doi: 10.1186/s13195-019-0569-x, PMID: 31881998 PMC6933667

[ref31] Tuncdemir S. N. Lacefield C. O. Hen R. (2019). Contributions of adult neurogenesis to dentate gyrus network activity and computations. Behav. Brain Res. 374:112112. doi: 10.1016/j.bbr.2019.112112, PMID: 31377252 PMC6724741

[ref32] Urbach A. Witte O. W. (2019). Divide or commit - revisiting the role of cell cycle regulators in adult hippocampal neurogenesis. Front. Cell Dev. Biol. 7:55. doi: 10.3389/fcell.2019.00055, PMID: 31069222 PMC6491688

[ref33] Urban N. Blomfield I. M. Guillemot F. (2019). Quiescence of adult mammalian neural stem cells: a highly regulated rest. Neuron 104, 834–848. doi: 10.1016/j.neuron.2019.09.026, PMID: 31805262

[ref34] Van Amerongen R. Bowman A. N. Nusse R. (2012). Developmental stage and time dictate the fate of Wnt/beta-catenin-responsive stem cells in the mammary gland. Cell Stem Cell 11, 387–400. doi: 10.1016/j.stem.2012.05.023, PMID: 22863533 PMC13155203

[ref35] Vancamp P. Gothie J. D. Luongo C. Sebillot A. Le Blay K. Butruille L.. (2019). Gender-specific effects of transthyretin on neural stem cell fate in the subventricular zone of the adult mouse. Sci. Rep. 9:19689. doi: 10.1038/s41598-019-56156-w, PMID: 31873158 PMC6927974

[ref36] Wang X. Kopinke D. Lin J. Mcpherson A. D. Duncan R. N. Otsuna H.. (2012). Wnt signaling regulates postembryonic hypothalamic progenitor differentiation. Dev. Cell 23, 624–636. doi: 10.1016/j.devcel.2012.07.012, PMID: 22975330 PMC3445042

[ref37] Wexler E. M. Paucer A. Kornblum H. I. Palmer T. D. Geschwind D. H. (2009). Endogenous Wnt signaling maintains neural progenitor cell potency. Stem Cells 27, 1130–1141. doi: 10.1002/stem.36, PMID: 19418460 PMC2782960

