# Supplementary material for: Exploring the dynamics of adult Axin2 cell lineage integration into dentate gyrus granule neurons
Source: Front Neurosci. 2024 Feb 21;18:1353142. doi: 10.3389/fnins.2024.1353142 (PMC10915230; doi:10.3389/fnins.2024.1353142)
Supplement: Supplementary file 1 [file Data_Sheet_1.pdf]

frontiers

Supplementary Material

Exploring the dynamics of adult *Axin2* cell lineage integration into dentate gyrus granule neurons

Khadijeh A. Sharifi[1,2], Faraz Farzad[1], Sauson Soldozy[1], Matthew R. DeWitt[3], Richard J. Price[4], Jason Sheehan[1], M. Yashar S. Kalani[1,2,5*], Petr Tvrdik[1,2*]

Departments of [1]Neurological Surgery, [2]Neuroscience, [3]Focused Ultrasound Cancer Immunotherapy Center, and [4]Biomedical Engineering, University of Virginia Health System, Charlottesville, VA, and [5]St. John's Neuroscience Institute and University of Oklahoma School of Medicine, Tulsa, OK

*** Correspondence:** tvrdik@virginia.edu and yashar.kalani@ascension.org

Supplementary Figures

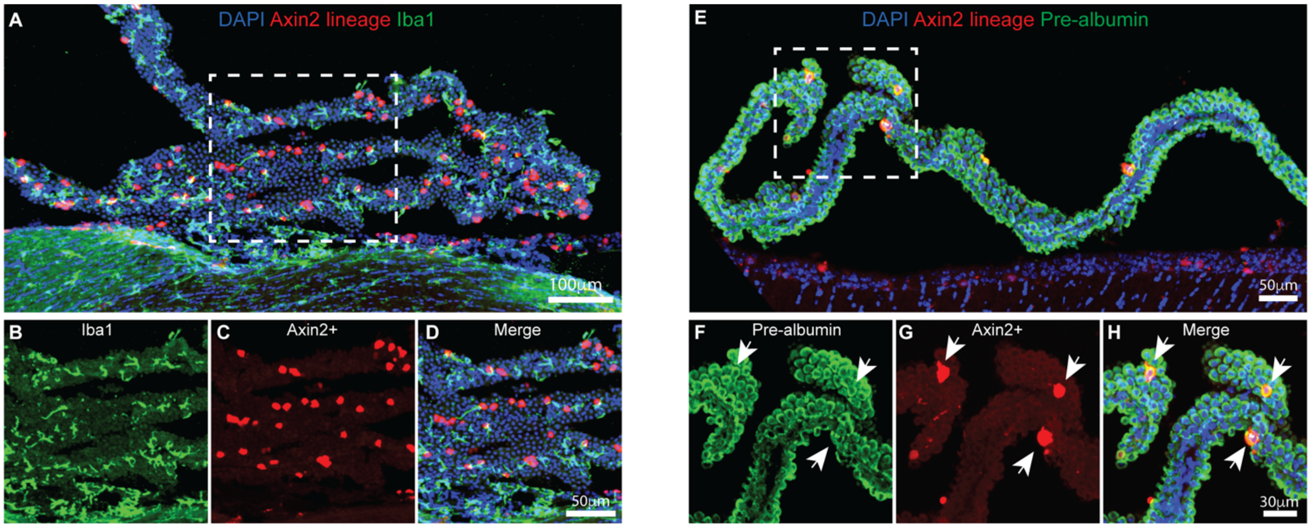

Supplementary Figure 1. Confocal analysis of Axin2 expression in the choroid plexus

A, Section through the choroid plexus from the 3rd ventricle one week after tamoxifen induction. The section was stained with anti-Iba1 antibodies and nuclear stain DAPI. Scale bar, 100 µm. **B**, Zoomed region, framed with the white dashed box in A, showing the Iba1 signal. **C**, The same field of view showing direct fluorescence of the Axin2 lineage marker tdTomato. **D**, Overlay demonstrating that Iba1 and tdTomato positive cells are mutually exclusive. Scale bar, 50 µm. **E**, A coronal choroid plexus section from same animal incubated with anti-Transthyretin (Prealbumin) and DAPI. Scale bar, 50 µm. **F**, Zoomed-in detail showing anti-Transthyretin staining. **G**, A corresponding field of view showing direct fluorescence of Axin2 - tdTomato. **H**, Overlay demonstrating that Axin2-tdTomato cells co-label with Transthyretin (Prealbumin), (denoted with white arrows). Scale bar, 30 µm.

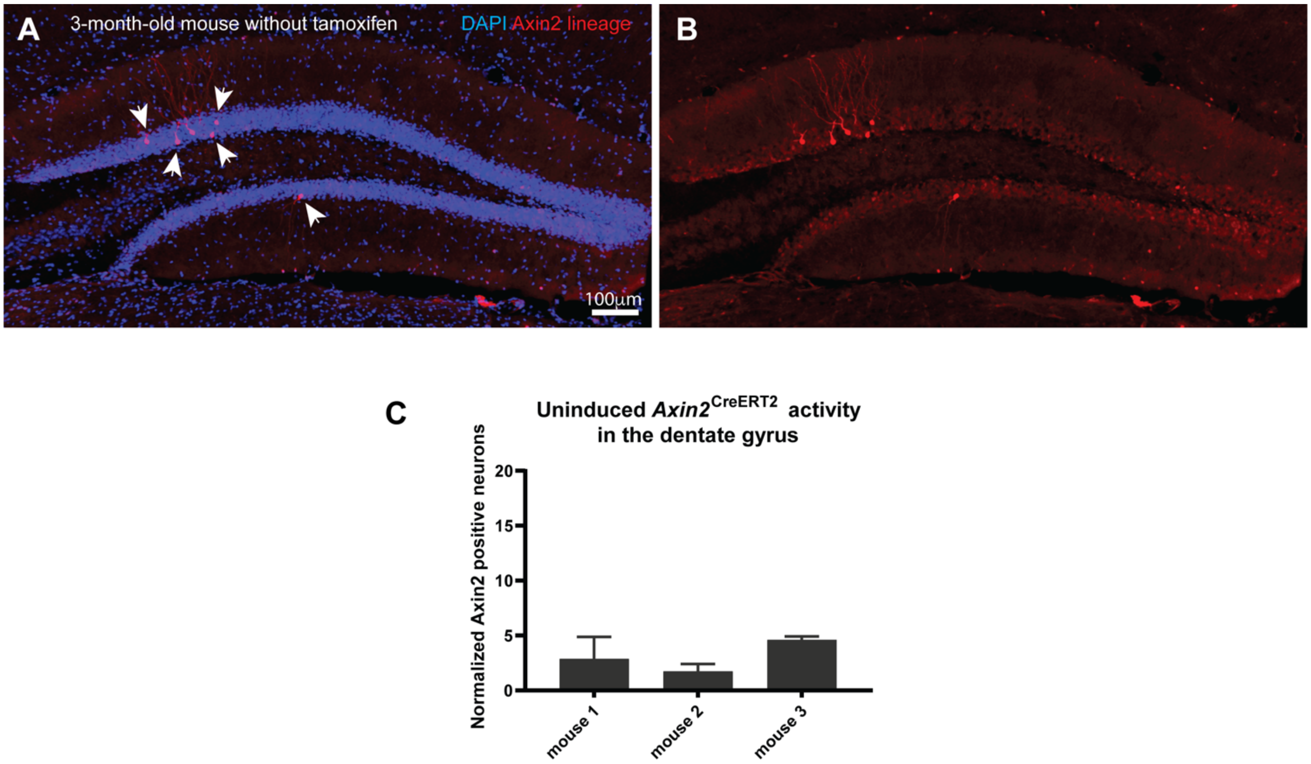

Supplementary Figure 2. Uninduced Axin2 CreERT2 activity in the adult hippocampus

A, Representative confocal image of the dentate gyrus (DG) showing Axin2 cell lineage (red) and DAPI (blue) staining in the brain sections from 3-month-old animals that were not induced with tamoxifen. The arrows point out the Axin2-positive cells. **B**, A corresponding single-channel image showing the tdTomato signal only, demonstrating Axin2 cell morphology consistent with neuronal differentiation. **C**, Graph showing the average density of Axin2 positive cells from 3-7 DG sections in 3 different animals (N = 13) without tamoxifen induction. These counts, normalized per 1000 DAPI positive nuclei, were 1.74 ± 0.67, 4.60 ± 0.32, and 2.87 ± 1.99, with the grand mean equal to 3.07 ± 1.44. This grand mean value was subtracted from nominal granule cell counts determined in Figure C-F, and the normalized adjusted values were plotted in Figure 2K.
